# Development of the Human Infant Intestinal Microbiota

**DOI:** 10.1371/journal.pbio.0050177

**Published:** 2007-06-26

**Authors:** Chana Palmer, Elisabeth M Bik, Daniel B DiGiulio, David A Relman, Patrick O Brown

**Affiliations:** 1 Department of Genetics, Stanford University School of Medicine, Stanford, California, United States of America; 2 Department of Microbiology and Immunology, Stanford University School of Medicine, Stanford, California, United States of America; 3 Department of Medicine, Stanford University School of Medicine, Stanford, California, United States of America; 4 Veterans Affairs Palo Alto Health Care System, Palo Alto, California, United States of America; 5 Department of Biochemistry, Stanford University School of Medicine, Stanford, California, United States of America; 6 Howard Hughes Medical Institute, Stanford University School of Medicine, Stanford, California, United States of America; Genome Institute of Singapore, Singapore

## Abstract

Almost immediately after a human being is born, so too is a new microbial ecosystem, one that resides in that person's gastrointestinal tract. Although it is a universal and integral part of human biology, the temporal progression of this process, the sources of the microbes that make up the ecosystem, how and why it varies from one infant to another, and how the composition of this ecosystem influences human physiology, development, and disease are still poorly understood. As a step toward systematically investigating these questions, we designed a microarray to detect and quantitate the small subunit ribosomal RNA (SSU rRNA) gene sequences of most currently recognized species and taxonomic groups of bacteria. We used this microarray, along with sequencing of cloned libraries of PCR-amplified SSU rDNA, to profile the microbial communities in an average of 26 stool samples each from 14 healthy, full-term human infants, including a pair of dizygotic twins, beginning with the first stool after birth and continuing at defined intervals throughout the first year of life. To investigate possible origins of the infant microbiota, we also profiled vaginal and milk samples from most of the mothers, and stool samples from all of the mothers, most of the fathers, and two siblings. The composition and temporal patterns of the microbial communities varied widely from baby to baby. Despite considerable temporal variation, the distinct features of each baby's microbial community were recognizable for intervals of weeks to months. The strikingly parallel temporal patterns of the twins suggested that incidental environmental exposures play a major role in determining the distinctive characteristics of the microbial community in each baby. By the end of the first year of life, the idiosyncratic microbial ecosystems in each baby, although still distinct, had converged toward a profile characteristic of the adult gastrointestinal tract.

## Author Summary

It has been recognized for nearly a century that human beings are inhabited by a remarkably dense and diverse microbial ecosystem, yet we are only just beginning to understand and appreciate the many roles that these microbes play in human health and development. Knowing the composition of this ecosystem is a crucial step toward understanding its roles. In this study, we designed and applied a ribosomal DNA microarray-based approach to trace the development of the intestinal flora in 14 healthy, full-term infants over the first year of life. We found that the composition and temporal patterns of the microbial communities varied widely from baby to baby, supporting a broader definition of healthy colonization than previously recognized. By one year of age, the babies retained their uniqueness but had converged toward a profile characteristic of the adult gastrointestinal tract. The composition and temporal patterns of development of the intestinal microbiota in a pair of fraternal twins were strikingly similar, suggesting that genetic and environmental factors shape our gut microbiota in a reproducible way.

## Abstract 3

Microarray profiling of the microbial communities of infant guts throughout the first year shows initial variation then convergence on the adult flora, providing new insight into this human ecosystem.

## Introduction

The adult human body typically comprises ten times more microbial cells than human cells, due largely to the extremely high density of microbes found in the human intestinal tract (typically 10^11^–10^12^ microbes/ml of luminal content). This microbial ecosystem serves numerous important functions for its human host, including protection against pathogens, nutrient processing, stimulation of angiogenesis, and regulation of host fat storage [1–7]. It is clear that this list is not yet complete; as this field of study expands, we are continually discovering new roles and relationships. Studies of gnotobiotic mice have been particularly enlightening, illustrating the essential role of the gastrointestinal (GI) microbiota in normal gut development [2,5]. In addition, numerous diseases in both adults and infants have known or suspected links to the GI microbiota, including stomach cancer [8], mucosa-associated lymphoid tissue lymphoma [9], inflammatory bowel disease [10,11], and necrotizing enterocolitis [12,13].

The composition of the adult GI microbiota has been intensely studied, using both cultivation and, more recently, culture-independent, small subunit (SSU) ribosomal DNA (rDNA) sequence-based methods [14]. The human colon ecosystem alone has been estimated to contain more than 400 bacterial species, belonging to a limited number of broad taxonomic divisions [15]. Members of the anaerobic genera *Bacteroides, Eubacterium, Clostridium, Ruminococcus,* and *Faecalibacterium* have typically been found to comprise a large majority of the human adult gut microbial community. Still, each adult's gut appears to have a unique microbial community, with a structure that remains stable on the time scale of months [3,15,16].

In contrast, the infant GI microbiota is more variable in its composition and less stable over time. In the first year of life, the infant intestinal tract progresses from sterility to extremely dense colonization, ending with a mixture of microbes that is broadly very similar to that found in the adult intestine [17]. Although the beginning and end points of this time course are well defined, the path between these points is poorly understood. There are conflicting reports in the literature regarding the composition of the neonatal GI microbiota and the factors that shape it. Several studies have reported that Bifidobacteria almost always dominate the GI microbiota of breast-fed infants by several weeks of age [17–20], while others find that they occur in only a small fraction of infants, or are not numerically dominant [21,22]. The effect of diet on the composition of the infant GI microbiota is also controversial—numerous studies have found a lower abundance of *Bifidobacteria* and a higher abundance of aerobic bacteria in the GI microbiota of formula-fed infants relative to breast-fed infants [20,21,23–25], yet other reports have found no such difference [26,27]. Mode of delivery has frequently been cited as one of the key factors that shape the infant microbiota [18,28,29]. The GI microbiota of infants delivered by caesarean section has been reported to differ from that of infants delivered vaginally, both in the timing of colonization and in composition [18,30–32], and in some cases, there are clearly traces of the maternal vaginal microbiota in the neonatal GI microbiota [33], yet the relative importance of mode of delivery on GI microbiota is unclear. Because of the increased incidence of GI problems in premature infants, the effect of gestational age has also been extensively studied. These studies have consistently shown that the microbiota of hospitalized, preterm infants differs from that of healthy, full-term babies [32,34–36]. Attempts to associate specific microbes with the occurrence of necrotizing enterocolitis, a condition with suspected bacterial etiology that is an important cause of morbidity and mortality in premature babies, have yielded mixed results [32,36]. Clearly, there is still much to be learned about the origins and development of the infant GI microbiota and its influence on health and disease.

We focused our study on describing the range of profiles that constitute a healthy infant GI microbiota in the hopes of discovering themes that govern its development, and in order to provide a detailed reference and a solid foundation for later studies examining the factors that influence the GI microbiota. Our study participants included 14 healthy, full-term babies, born to 13 healthy mothers (thus including one set of fraternal twins) (Table 1). Stool samples were collected according to a prescribed schedule, beginning with the first stool produced after birth: samples were collected daily at first and then with decreasing frequency over the course of 1 y, with additional sampling around key events (e.g., introduction of solid food and administration of antibiotics), yeilding an average of 26 stool samples per baby (Table 2). In addition, stool samples were collected from parents and siblings, and vaginal swabs and breast milk were collected from the mothers. We analyzed the microbiota of each of these specimens using a newly developed SSU rDNA microarray designed to give nearly comprehensive coverage of known SSU rDNA species. A subset of these samples was also analyzed by SSU rDNA clone library sequencing, for the purposes of calibrating and validating our microarray results.

**Table 1:**
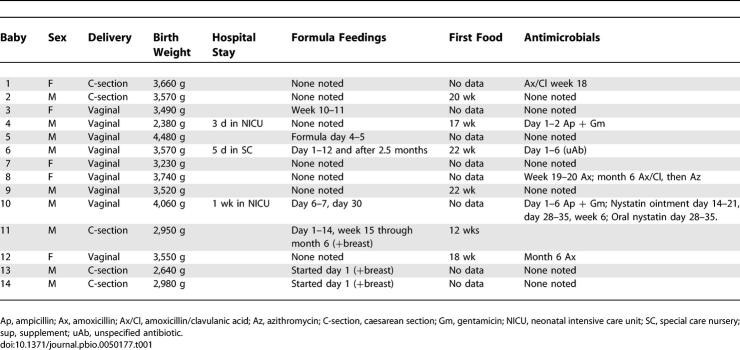
Relevant Characteristics of the Infants in This Study

**Table 2:**
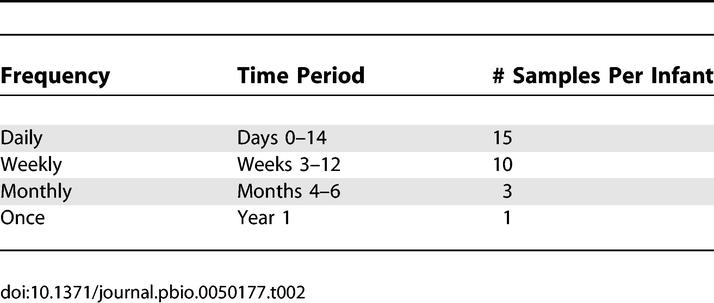
Infant Stool Sample Collection Schedule

## Results

### Comparison of Microarray- and Sequence-Based Bacterial Population Profiles

To survey the composition of our sample set and to provide a basis for quantitative calibration of the microarray results, we created a reference pool by combining equal amounts of amplified SSU rDNA from each PCR-amplifiable sample (except for samples collected when the infants were ≥1 y old). We obtained 3,458 high-quality clone sequences from a library constructed from this pool, and taxonomically assigned each sequence using Ribosomal Database Project's Classifier [37]. The taxonomic distribution of these sequences is summarized in Table 3.

**Table 3:**
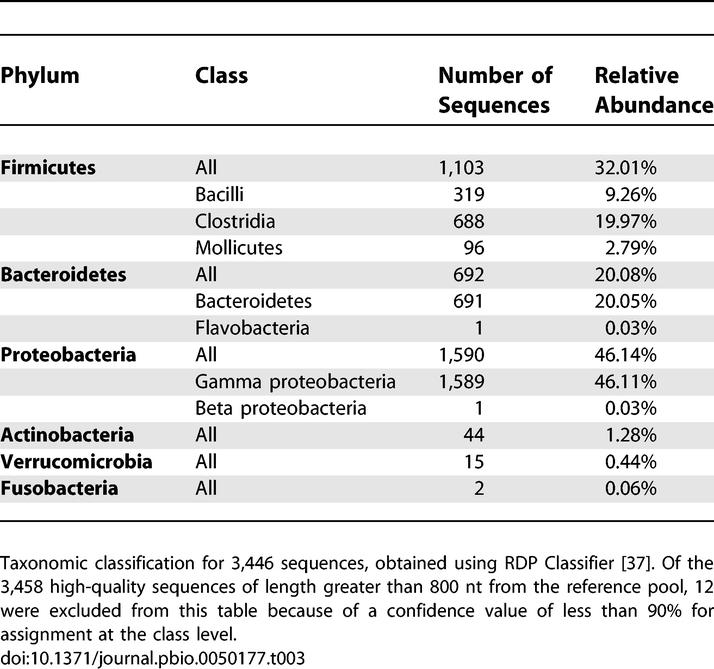
Reference Pool Composition

To assess the performance of our new microarray design relative to SSU rDNA sequencing, we sequenced SSU rDNAs amplified from each of 12 individual biological samples obtained in this study, selected for their diverse profiles by 16S rDNA microarray analysis. This study set included DNA extracted from eight baby stools, two maternal stools, one vaginal swab, and one breast milk sample. For each of these samples, we amplified SSU rDNA sequences using the same PCR primers that were used in the microarray analysis, then cloned and sequenced several hundred (mean = 342) of the amplified products for a total of 4,100 sequences.

We focused our comparison at levels 2, 3, and 4 of the prokaryotic multiple sequence alignment (prokMSA) hierarchy, which very roughly correspond to the phylum, class, and order levels in the classical taxonomic hierarchy. At these broader levels, most sequences are expected to have homology to at least one probe in our current microarray design, and rDNA sequences can generally be unambiguously classified. Microarray-based relative abundance estimates were obtained for 2,149 species and taxonomic groups by integrating data from all probes that represented any subset of the class in question, as fully described in Materials and Methods. Sequence-based estimates were obtained by taxonomically classifying each sequence by assigning the prokMSA operational taxonomic unit (OTU) code of the best BLAST match in the 2004 prokMSA database of 86,453 SSU ribosomal RNA (rRNA) gene sequences [38] (Datasets S1 and S2). Although the relative abundance of a bacterial species cannot be precisely determined from its proportional representation in a pool of amplified rDNA sequences, we expect that such estimates should be accurate within an order of magnitude and usually within a few-fold [39–41], based on previous studies that compared abundance levels estimated from sequencing SSU rDNA amplicons with counts based on in situ hybridization.

**Figure 1:**
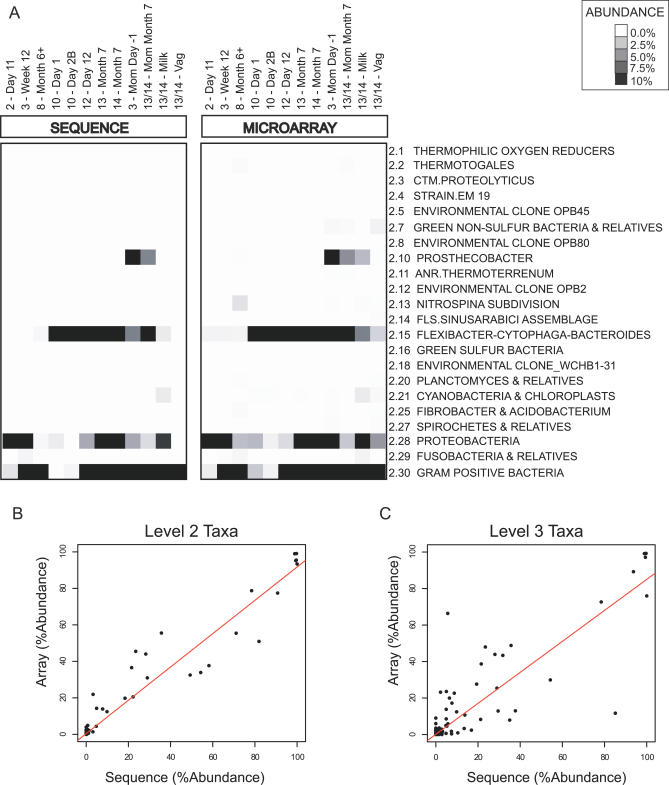
Comparison of Microarray- and Sequencing-Based Community Profiles Microarray-derived and sequencing-derived data estimates of taxonomic group abundance are compared for 12 biological samples. (A) Abundance estimates for all prokMSA level 2 taxa measured on the array are compared. Each column represents a single biological sample and each row corresponds to a single taxonomic group, identified (to the right of each row) by its numerical prokMSA OTU code, along with the roughly corresponding conventional name for the group. (B) Comparison of sequence-based and microarray-based relative abundance estimates for level 2 taxonomic groups in 12 samples (same as in [A]). The *x*-axis represents the relative abundance as estimated by the frequency of clones from a given taxonomic group, and the *y*-axis represents the relative abundance as estimated by microarray profiling. (C) Same as (B) for level 3 taxonomic groups.

Overall, the microarray results were very similar to those obtained by sequencing, both qualitatively and quantitatively. Figure 1A shows the comparison of the community profiles of each of the 12 samples derived from our microarray analysis and by sequencing, for each taxonomic group at level 2 of the prokMSA taxonomic tree. Note that the levels (e.g., level 2) in the prokMSA taxonomy do not have a consistent correspondence with the levels (e.g., phylum) in the classical taxonomic hierarchy, and thus some of the conventional names associated with prokMSA level 2 groups can appear somewhat incongruous. Both the sequence analysis and the microarray analysis showed that the samples were dominated by a limited number of taxonomic groups—99% of the 4,100 sequences were encompassed by just three of the 22 level 2 prokMSA divisions: 2.15 *(Flexibacter-Cytophaga-Bacteroides),* 2.28 (Proteobacteria), and 2.30 (Gram-positive bacteria [including Firmicutes and Actinobacteria]), and the remaining 1% belonged to groups 2.10 *(Prosthecobacter),* 2.29 (Fusobacteria), or 2.21 (Cyanobacteria and Chloroplasts). As shown in Figure 1B and 1C, the population profiles obtained by microarray and sequencing analysis were also quantitatively similar—the Pearson correlation of the microarray- and sequencing-based estimates of relative abundance for the 12 samples was 0.97 at prokMSA taxonomic level 2 (Figure 1B), 0.89 at level 3 (Figure 1C), and 0.80 at level 4 (unpublished data).

### Absolute Quantification of Bacteria

**Figure 2:**
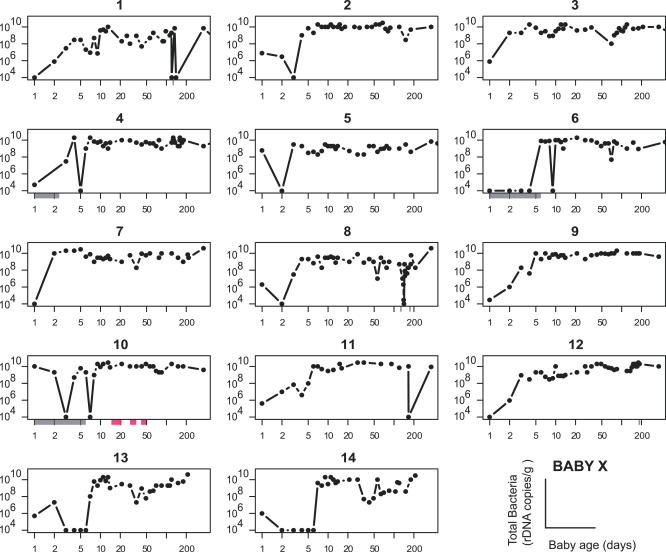
Variation in the Overall Density of Fecal Bacteria during the First Year of Life. For each baby sample, bacterial abundance was estimated by TaqMan real-time PCR with universal bacterial primers. Estimated rRNA gene copies per gram of feces (*y*-axis) are plotted as a function of days of life (*x*-axis). Both axes are on a logarithmic scale. Abundance measurements are truncated on the lower end at the value corresponding to the 95th percentile of the extraction (negative) controls (copy number corrected by median stool mass). Episodes of antibacterial or antifungal (nystatin) treatment are indicated on the temporal axis by gray or pink bars, respectively (see Table 1 for additional information).

We estimated the overall density of bacteria in each sample by a real-time quantitative PCR (qPCR) assay, using a broad-range bacterial primer and probe set (see Materials and Methods). We used the total number of rRNA gene copies (typically about five per genome [42]) per gram of stool, as estimated by this assay, to approximate the total density of bacteria. As shown in Figure 2, the total number of rRNA gene copies was relatively unstable throughout the first week of life, then persisted in most babies in the range of 10^9^ to 10^10^/g of stool (wet weight). Although there was no clear effect of method of delivery on the timing of the colonization, it is noteworthy that babies 13 and 14 (the dizygotic twins), who were the only babies delivered by a planned caesarean section, and thus without rupture of the amniotic membrane and exposure to maternal birth canal microbiota during labor or delivery, had low bacterial counts (<10^8^ rRNA gene copies/g) until the seventh day of life.

### Overview of Microarray-Based Bacterial Population Profiles

**Figure 3:**
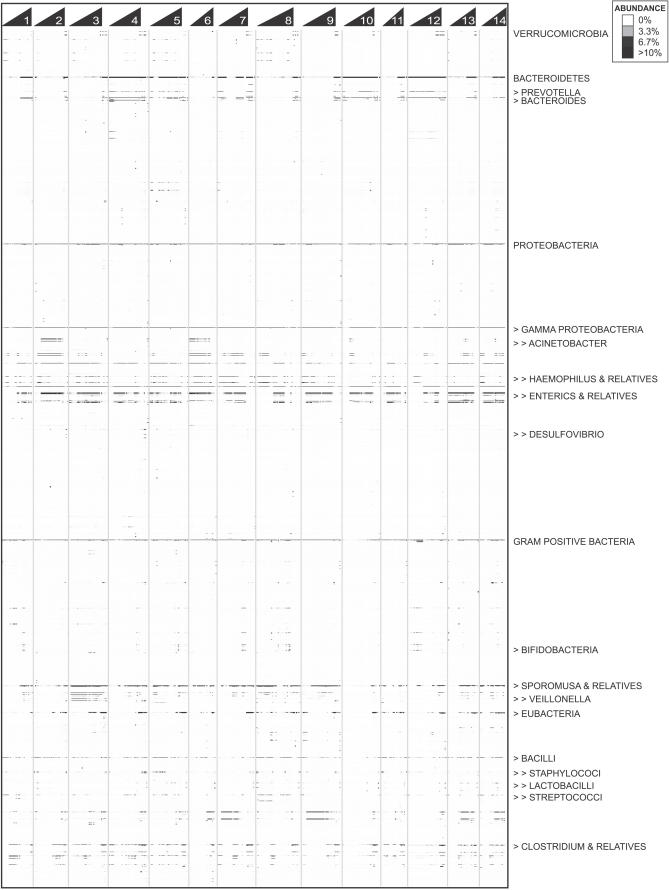
Overview of Microbial Community Profiles of All Samples Each column (*n* = 430) represents one biological sample and each row (*n* = 2,149) represents one taxonomic group or species. Samples are organized in temporal order, beginning with birth at left and any maternal- or other family-derived samples at the right of each time course. Wedges above columns are numbered according to baby identifier. Rows (taxa) are sorted by their numerical codes so that subgroups of a given group lie directly below the more general group (e.g., 2.15, then 2.15.1, then 2.15.1.1). The symbols “>” and “> >” are added to the names of labeled taxonomic groups that are subgroups, at level 3 or level 4, respectively, of a labeled level 2 taxonomic group. Increasing darkness of the grayscale corresponds to higher estimated relative abundance.

We analyzed the bacterial composition of 430 samples—363 infant stool samples, 43 adult stool samples, two sibling stool samples, 12 breast milk samples, and ten maternal vaginal swabs—by hybridization to the DNA microarray developed in this study. By combining information across multiple probes (see Materials and Methods), we obtained relative abundance estimates for 2,149 nested taxonomic groups and species in each of these samples (All probes are listed in Dataset S3; All taxa are listed in Dataset S4). As shown in Figure 3, the phylum-level diversity in the stool samples analyzed in this study was extremely limited. The vast majority of samples were dominated by just three of the 22 level 2 bacterial groups represented by our microarray: 2.15 *(Flexibacter-Cytophaga-Bacteroides),* 2.28 (Proteobacteria), and 2.30 (Gram-Positive Bacteria [Firmicutes and Actinobacteria]). A second major finding was the remarkable degree of interindividual variation in the colonization process. Although the taxa that populate the infant GI tract were limited at the broadest levels, each baby was distinct in the combination of microbial species that it acquired and maintained, and in the precise temporal pattern in which those species appeared and disappeared. *Bacteroides,* for example, dominated the early microbiota of some babies but were virtually absent at this stage in other babies. A third striking feature of this dataset was the relative stability of the microbial populations over time—even early in the course of the colonization of the infant GI tract, most taxonomic groups persisted over intervals of weeks to months.

The main dimensions of variation among the colonization profiles of different taxonomic groups were timing of colonization and temporal stability. Consistent with previous studies [28,35,43,44], the earliest colonizers were often organisms predicted to be aerobes (e.g., *Staphylococcus, Streptococcus,* and Enterobacteria), whereas the later colonizers tended to be strict anaerobes (Eubacteria, and Clostridia)*.* The *Bacteroides* varied greatly from baby to baby in the timing of their first appearance, but were consistently present to some degree in nearly all babies by 1 y. Several other taxa, including *Prevotella, Acinetobacter, Desulfovibrio, Veillonella,* and *Clostridium perfringens,* tended to appear only transiently, sometimes appearing and disappearing repeatedly within a baby's first year of life.

### Similarities and Differences among Population Profiles

**Figure 4:**
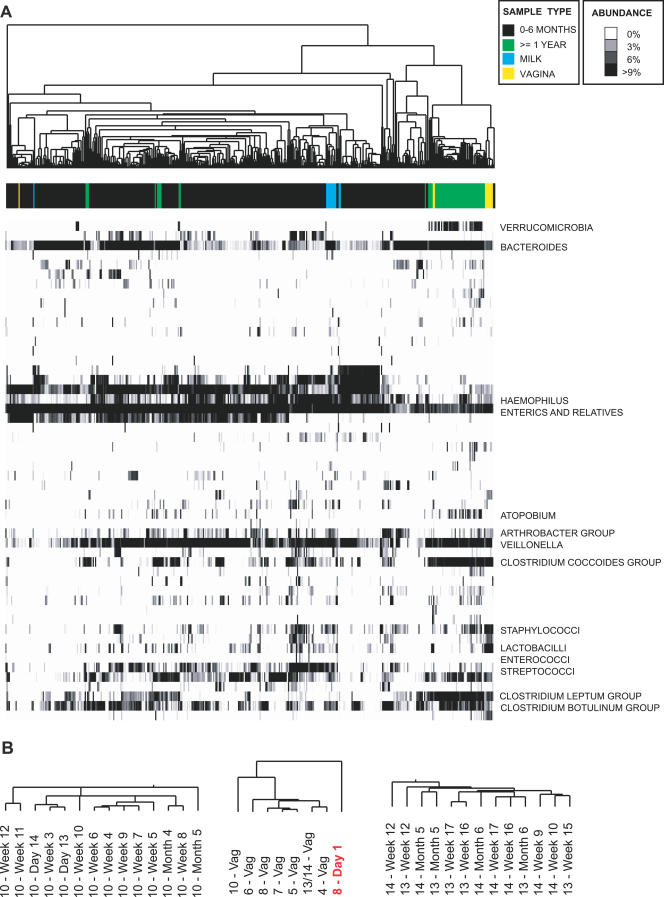
Clustering of Samples Based on Population Profiles of Most Common and Abundant Taxa (A) Each column (*n* = 430) represents one biological sample, and each row (*n* = 52) represents one level 4 taxonomic group or species for which two or more samples had relative abundance estimates greater than 1%. All samples, including stool samples from the subject infants, parents, and siblings, as well as milk and vaginal samples, are represented. Samples were clustered by centered Pearson correlation, so that columns representing the most similar samples are grouped together, whereas taxonomic groups (rows) are numerically sorted rather than clustered. Increasing darkness of the grayscale corresponds to higher estimated relative abundance. Values are log_2_ of relative abundance. (B) Selected clusters illustrating that (1) profiles from early baby stool samples cluster by baby, (2) very early baby samples cluster with maternal samples, and (3) samples from the pair of fraternal twins cluster together and intermingle.

We explored the similarities and differences in the composition of all of our samples by hierarchically clustering the 430 samples based on their similarity with respect to their abundance profiles for the set of 53 prokMSA level 4 taxonomic groups that had at least two samples with a relative abundance estimate greater than 1%. The clustering pattern, as reflected in the dendrogram at the top of Figure 4, highlights several critical features of the colonization program, and shows that the stool microbiota of babies 1 y of age and older is distinctly different from that at earlier ages and much more similar to that of adults. Prior to 6 mo of age, stool samples tended to cluster by baby, indicating that the differences from baby to baby are much greater than the changes over periods of weeks or months in the composition of any individual baby's microbiota. There were two notable exceptions to this baby-specific clustering. First, samples from the first few days of life often clustered away from the rest of a given baby's samples, sometimes clustering with other very early samples and sometimes with samples from other sites (e.g., baby 8 day 1 with vaginal samples). Second, samples from babies 13 and 14, who are fraternal twins, tended to intermingle. Figure 4B shows examples of several of the clustering patterns described above.

Most of the breast milk and maternal vaginal samples clustered perfectly by anatomic site of origin. As expected, all but one of the vaginal samples were overwhelmingly dominated by lactobacilli, with Staphylococci*, Bacteroides,* Clostridia, and *Veillonella* among the groups variably present as minority constituents. The vaginal sample from one of the mothers (mother of baby 11) had a distinctly different population profile, dominated instead by members of the Gamma Proteobacteria group. The microbial populations found in the milk samples were diverse, often including mixtures of enterics and species of *Bacteroides, Pseudomonas, Haemophilus, Veillonella,* and *Streptococcus*.

**Figure 5:**
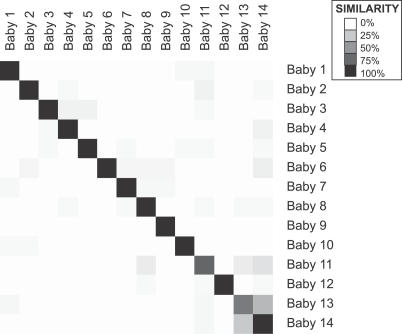
Similarity of Microbiota between Babies For each pair of samples, we calculated the nearest-neighbor samples according to Pearson correlation of the level 4 relative abundance profiles. For each baby, we then computed what percent of nearest-neighbor samples came from each baby. The shade of grey indicates the percent of samples from baby Y (column) that were nearest neighbors of the samples from baby X (row) such that rows add to 100%.

In order to compare the infants more systematically, we determined the nearest-neighbor sample for each sample as measured by the Pearson correlation of level 4 relative abundance estimates. Using this metric, the nearest-neighbor sample of any given baby sample was usually another sample from the same baby—the average percentage of samples from a given baby for which the most similar sample was from the same baby was 82%. Figure 5 summarizes this analysis and illustrates the interesting finding that by this measure, the most similar pair of babies by far was babies 13 and 14—fraternal twins raised in the same environment—8 of 23 (35%) of baby 13′s nearest-neighbor samples were from baby 14 (the next most similar pair was babies 11 and 14, at 17%).

### Temporal Trends

**Figure 6:**
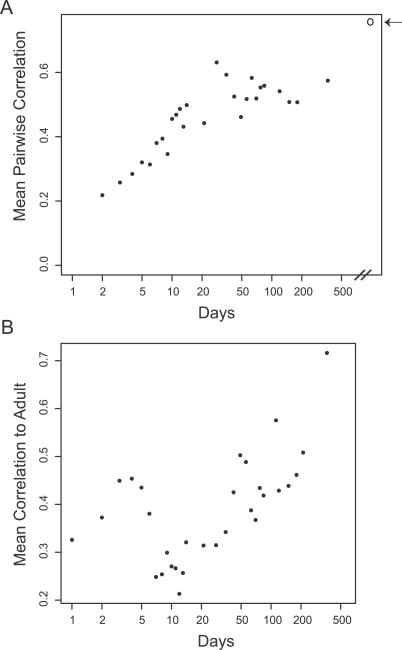
Temporal Patterns in Average Pairwise Similarity of Infant Stool Microbiota Profiles (A) Similarity between infants over time. For each time point for which at least six babies were profiled, we calculated the mean pairwise Pearson correlation between the level 4 taxonomic profiles of all babies at that time point. The mean pairwise Pearson correlation between these profiles in 18 adult participants in this study (nine males and nine females) is also shown (open circle indicated by the arrow). (B) Progression towards adult-like flora over time. For each time point for which at least four babies were profiled, we calculated the mean Pearson correlation between the level 4 taxonomic profiles of all babies at that time point and a “generic adult” profile. The generic adult profile is the centroid of 18 (nine male and nine female) adults (parents in this study).

The similarity of the microbial community profiles of stool samples from babies 1 y and older to each other and to those of the adult stool samples suggested that the infant GI communities converged over time toward a generalized “adult-like” microbiota. We explored this phenomenon by calculating, for each age interval, the average pairwise Pearson correlation of the population profiles of all infant samples collected at that age. As shown in Figure 6A, this analysis revealed that as time progressed, the babies' microbiota consistently converged toward a common profile. We also calculated, for each time point, the average correlation of infant samples at that time point to a generalized adult profile (centroid of 18 adult samples—nine fathers and nine mothers from this study). This analysis, shown in Figure 6B, confirmed that the profile toward which the infants' microbiota converges is similar to that of adults, and highlighted an apparent tendency for a population rearrangement to occur around 5 d after birth. Notably, the infants' GI microbiota was not significantly more similar to that of their parents than to that of other adults, as measured by the Pearson correlations of their level 4 taxonomic profiles (mean baby–parent correlation of 0.55 for within family, versus 0.62 between families for nine “triads” of contemporaneously obtained samples from baby, mother, and father obtained at 1–1.5 y of age).

**Figure 7:**
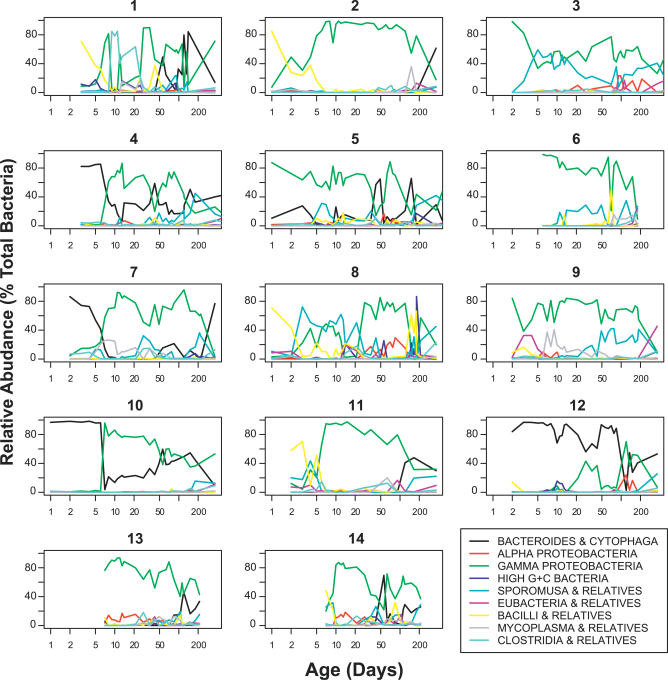
Temporal Profiles of the Most Abundant Level 3 Taxonomic Groups Level 3 taxonomic groups were selected for display if their mean (normalized) relative abundance across all baby samples was greater than 1%. The *x*-axis indicates days since birth and is shown on a log scale, and the *y*-axis shows estimated (normalized) relative abundance. For some babies, no values are plotted for the first few days because the total amount of bacteria in the stool samples collected on those days was insufficient for microarray-based analysis.

To visualize the temporal patterns in the particular phylogenetic groups that populate the infant gut, we charted the relative abundance of the nine level 4 taxonomic groups that had a mean relative abundance of 1% or greater over time in each infant (Figure 7). This analysis enabled us to identify common themes and interesting differences among the colonization profiles of these babies. First, we observed that “uneven” populations (populations heavily dominated by a single taxonomic group) were common in the first several weeks but rare later in the time courses. Another notable feature in the temporal program of many of the babies was the occurrence of one or more dramatic shifts in the population structure—such shifts were frequently stabilized within one sampling interval. We were unable to identify any specific age or signal event consistently associated with such transitions, although the transition to an “adult-like” profile often followed the introduction of solid foods.

Several of the babies were treated with antibiotics either in the neonatal period (day 0–28) or in the later months (see Table 1 and Figure 2 for more details). In some cases, the treatment was associated with a striking alteration in the density or composition of the GI microbiota. For example, baby 8 received two courses of amoxicillin, one at 4 mo and one at 6 mo. In both cases, both the total density of bacteria (Figure 2) and the community composition were dramatically altered (Figures 3 and 6). Indeed, in this baby, the bacterial density in fecal samples decreased so much during the antibiotic courses that we were unable to amplify sufficient SSU rDNA for microarray analysis, so we could only compare the populations before and after the antibiotic course. However, we did not identify any consistent consequences of antibiotic treatment.

### Microarray Validation Experiments

The results of both the sequence analysis of the reference pool and the microarray data analyses indicated that Bifidobacteria were only minor components of the population—a result at odds with the conventional wisdom [20,21,26]. The primers we used for broad-range PCR amplification of the reference pool (the source of the sequences) and samples for microarray analysis were potentially suboptimal for amplification of Bifidobacteria [21,26] due to three mismatches in the rDNA sequence of Bifidobacterium longum to the forward primer 8F used in this study. A survey of the 5′ sequences of full-length SSU rDNA genes showed that Bifidobacteria are outliers in their divergence from the generally conserved 8F primer sequence. We therefore carried out two independent analyses to determine whether and how the quantitative estimates of Bifidobacteria from the microarray hybridization results would need to be adjusted. First, we quantitatively evaluated the relative efficiency with which the 8F/1391R primer pair amplified SSU rDNA from two Bifidobacteria species—Bifidobacterium longum and *Bifidobacterium infantis—*compared to a set of three diverse common fecal bacteria—*Escherichia coli, Clostridium perfringens, Bacteroides fragilis*—all of which have SSU rDNA sequences with one or more mismatches to the 8F/1391R PCR primer sequences. Using a range of stoichiometric mixtures of chromosomal DNA extracted from these species, we found that after 20 cycles (the number of cycles used for our microarray analyses and for amplification of the reference pool prior to sequencing), efficiency of amplification of the two Bifidobacterial species' DNA was consistently 8-fold lower than that of the three other species, all of which amplified with nearly identical efficiencies (unpublished data). This result suggests that both the reference pool sequencing results and the microarray-based quantitation underestimated the abundance of the Bifidobacteria group by a factor of eight. Second, we used a real-time qPCR assay with a primer pair and probe optimized for detection of Bifidobacteria to obtain an independent estimate of the abundance of Bifidobacteria in each sample. The results confirmed the finding from the microarray analysis that Bifidobacteria were almost always only minor constituents of the fecal microbiota of both the infants and adults in our study population (Dataset S5 and Figure S1).

The majority of bacterial species identified in our sample set were previously reported constituents of the human GI microbiota. There were, however, a number of cases in which the microarray results indicated the presence of a bacterial species or group that was both unexpected and not represented in our sequenced reference pool. We investigated several of these cases using independent assays. For 12 of the suspect species/taxa, we used the cognate group-specific primers in a PCR assay applied to most or all of the samples in which the suspect species/taxa appeared to be present based on the microarray results, as well as a small set of samples in which the suspect species was not detected by the microarray. In one case, that of *Sutterella wadsworthia,* sequencing of the species-specific PCR product confirmed its presence. In seven of 12 cases, none of the array positive (or negative) samples yielded an amplified product in the PCR analysis. For four remaining cases, the ostensibly species-specific PCR assay yielded an amplified product of the expected size, but the clones sequenced from this product did not correspond to the expected species. We further investigated these four cases by sequencing a clone library obtained by amplification with the same broad-range primers that were used in preparation for microarray analysis. Although the sequencing did not confirm the presence of any of the four questionable species/taxa, it provided strong evidence for a major source of false-positive hybridization signals. Specifically, in three of the four cases, we identified a relatively abundant species whose rDNA sequence was sufficiently similar to the probe sequence that it was likely to account for the observed signal. In one case *(Legionella pneumophila),* which was predicted to be present at approximately 1%, we were unable to identify any candidate species that could account for the hybridization signal (i.e., none with best BLAST matches scores ≥30), among our set of 192 sequences. Since our power to detect a species present at a partial abundance of 1% was only 85%, it remains possible that this species, or another species with a similar SSU rDNA sequence, could have been present at a low abundance in the suspect samples.

### Detection and Quantification of Fungi and Archaea

Both our DNA extraction and rDNA amplification methods were optimized for bacteria and suboptimal for eukaryotes and archaea, thus we separately tested for the presence and abundance of fungi or archaea by means of qPCR assays with broad specificity for the respective taxonomic groups. Based on our qPCR analysis, fungi were intermittently detectable in stool samples at relatively low abundance (10^4^–10^6^ rRNA genes/g fecal wet weight), persisting for varying durations in individual babies, through the first year of life. One of the babies in this study (baby 10) was noted to have a diaper rash, as well as oral thrush, both of which are commonly caused by a fungus *(Candida),* and which were treated with an antifungal agent (nystatin). The qPCR analysis detected especially high levels of fungal rDNA in stool samples from this baby, particularly during the period in which these findings were described. This baby's mother also had notably high levels of fungal SSU rDNA sequences in her prenatal vaginal swab sample, but not in her “day 0” stool sample.

The prevalence of archaea was considerably lower and more variable than that of fungi or bacteria; qPCR analysis detected archaeal rRNA genes (in the range of 10^3^–10^6^ rRNA genes/g) in only seven babies during their first year of life, and in four of these babies, they were detected in only a single sample. In these babies, archaea appeared only transiently, and almost exclusively in the first few weeks of life; they were detected in only one infant after the fifth week of life. Limited analysis of archaeal sequences amplified from the three maternal stool samples that tested positive for archaea (mothers 4, 9, and 12) revealed a predominance of Methanobrevibacter smithii (7/8 archaeal clones identified, including at least one clone from each mother), with one additional (uncultured) archaeal phylotype. Results of qPCR analysis of fungi and archaea are included in Dataset S5 and shown graphically with bacterial qPCR results in Figure S2.

## Discussion

The microbial colonization of the infant GI tract is a remarkable episode in the human lifecycle. Every time a human baby is born, a rich and dynamic ecosystem develops from a sterile environment. Within days, the microbial immigrants establish a thriving community whose population soon outnumbers that of the baby's own cells. The evolutionarily ancient symbiosis between the human GI tract and its resident microbiota undoubtedly involves diverse reciprocal interactions between the microbiota and the host, with important consequences for human health and physiology. These interactions can have beneficial nutritional, immunological, and developmental effects, or pathogenic effects for the host [2,5,7,18,45].

This study began with the development of a DNA microarray with nearly comprehensive coverage of the bacterial taxa represented in the available database of SSU rRNA gene sequences. Our microarray design and experimental methods were based on lessons learned in the validation of a less comprehensive SSU rDNA microarray [46]. These previous experiments enabled us to optimize our methods for computational prediction of SSU rDNA hybridization behaviors, and to develop an experimental protocol that maximized hybridization specificity. The excellent concordance in the measurements of individual taxa determined using the new microarray design in comparison with sequencing results from corresponding SSU rDNA clone libraries (Figure 1) suggests that these design principles hold true for this platform across a diversity of taxa and give us confidence in both the comprehensiveness and accuracy of the results obtained with our new microarray probe set. It is important to note, however, that our methods of array design and analysis are imperfect and still evolving. Several of the unexpected species predicted by the microarray to be present in one or more samples could not be corroborated by sequencing. In most of these cases, sequence analysis of the sample(s) in question revealed that low-level cross hybridization of a highly abundant species was responsible for the false-positive prediction, a result that will be taken into consideration in future rounds of array design and analysis.

We used this microarray in a detailed, systematic, and quantitative study of bacterial colonization of the newborn human GI tract. We used freshly collected stool samples as surrogates for samples taken from the lumen and mucosa of the colon. Although there are undoubtedly differences in the population profiles of stool samples and corresponding mucosa, we found in a previous study that the profiles are nonetheless remarkably consistent—sufficiently so that individual stool samples can readily be matched to colonic biopsy samples from the same individual, based on the similarity in their bacterial profiles [15,46]. Thus, we believe that the results of our temporal analysis of the bacterial populations in infant stool samples provide a useful window on the resident colonic microbiota.

In view of the importance of the symbiosis between human host and gut commensals for both human host and microbial colonist, it would be easy to imagine that the program of microbial colonization of the neonatal GI tract would have evolved under strong selective pressure, acting on both the intestinal niche and its microbial colonists, to be highly deterministic and stereotyped. We might have expected that a highly restricted group of co-evolved commensals would be exceptionally well adapted to this environment and consistently dominate the colonization process in a stereotyped fashion. Indeed, the bacteria that we found in infant and adult feces, presumably reflecting the colonic microbiota, were largely restricted to only a small subset of the bacterial world—Proteobacteria, *Bacteroides,* Firmicutes, Actinobacteria, and Verrucomicrobia. Yet, surprisingly, we found that in the first days to months of life, the microbiota of the infant gut, and the temporal pattern in which it evolves, is remarkably variable from individual to individual. The seemingly chaotic progression of the early events in colonization, and the similarity in bacterial composition of some early infant samples to breast milk or vaginal swabs, suggests that the bacterial population that develops in the initial stages is to a significant extent determined by the specific bacteria to which a baby happens to be exposed. Notably, these maternal “signatures” did not persist indefinitely, as evidenced by our failure to find a significantly higher correlation of the overall taxonomic profiles of baby/parent pairs from the same household versus different households.

An important exception to the tale of individuality and uniqueness in the early profiles was the remarkable similarity of the temporal profiles of the fraternal twins (babies 13 and 14) (Figures 4 and 5). These twins shared both a common environment and approximately 50% genetic identity, making it impossible to determine from this study to what degree each of these commonalities is responsible for their similar colonization patterns. However, evidence from this and other studies suggests that the shared environment is a major factor. One argument in favor of this view is the lack of comparable similarity in the microbial communities of other pairs that also share 50% genetic identity, including mother:baby, father:baby, and sibling:baby (unpublished data), although this dissimilarity may be due in part to their differing stages in development. Another argument in favor of a strong environmental influence is the coincidental transient appearance of specific organisms in both twins—it is hard to imagine that the appearance of a particular microbe on a particular day could be genetically programmed. Our final argument rests on evidence from a previous study that the microbiota of genetically equivalent families from a cross of inbred mice was more similar among members of the same “household” (mother and offspring who share a cage) than between households [1].

The definition of a “healthy” intestinal microbiota encompasses a remarkable diversity of community profiles in the first 6 mo of life. Although diverse and idiosyncratic in the early months, these microbial communities became progressively more similar to one another (Figure 6A), converging toward a generic adult-like profile (Figure 6B) characterized by a preponderance of *Bacteroides* and Firmicutes, common occurrence of Verrucomicrobia, and very low abundance of Proteobacteria and aerobic Gram-negative bacteria in general. We hypothesize that the earliest colonization events are determined to a large extent by opportunistic colonization by bacteria to which a baby is exposed in its environment. Common environmental exposures are likely to include the maternal vaginal, fecal, or skin microbiota, as is suggested by the observed similarity of some infants' early stool microbiota to these samples, which is consistent with previous evidence of vertical transmission of microbes [33,47,48]. The diversity and variation would thus reflect the corresponding individuality of these accidental exposures. Over time, however, the fitness advantage of the taxa that typically dominate the adult colonic microbiota apparently overcomes the initial advantage of early-colonizing opportunists that are less well adapted to the intestinal environment. In addition, progressive changes in the gut environment, due to intrinsic developmental changes in the gut mucosa, transition to an “adult” diet, and the effects of the microbiota itself [44,49–51], may impose increasingly stringent selection for the most highly adapted bacteria. Thus, despite the unexpectedly chaotic early months, the establishment of the gut ecosystem in human infants turns out after all to follow a conserved, conventional program.

The transformation of the intestinal microbiota to an adult-like pattern implicitly involved replacement of species found in infants, but rarely in adults, with species characteristically found in the adult colon. One potential driving force for such a demographic change might be that the adult-like community members eventually dominate by virtue of their greater ability to establish themselves stably and irreversibly once they colonize a host. We looked for evidence of this differential “stickiness” by comparing the autocorrelations over time of the abundance of each “species” (see Materials and Methods). We found no clear evidence that the species characteristic of adult microbiota were able to establish more intrinsically stable colonization than the species characteristic of infant microbiota.

We and others have found that the individual-specific characteristics of the bacterial microbiota of adults are stable, in the sense that they remain consistently more similar within an individual over time than between individuals, for periods of a year or more, and one of the striking results of this study was the identification of relatively stable, individual-specific patterns of bacterial colonization even in the first weeks and months of life. These observations raised the interesting possibility that opportunistic colonization events in early infancy might play a significant role in defining the distinct characteristics of the same individual's microbiota into adulthood. We looked for evidence of this by comparing the intraindividual and interindividual correlations of bacterial profiles at 1 or 2 mo and 1 y, and found no significant difference (unpublished data). Thus, although these results certainly do not exclude the possibility that early colonization events play an important role in determining the adult microbiota, there does not appear to be a strong, direct correlation between the two.

Our results and conclusions differ considerably from many previous reports in several respects. One notable discrepancy between our studies and many others was the relatively low frequency and abundance of Bifidobacteria in the fecal microbiota at any age from birth to adulthood. Bifidobacteria have received disproportionate attention, in part because of their reputed beneficial effects, and many studies have reported (and reviews have repeated) that the microbiota of breast-fed infants is dominated by Bifidobacteria [17–19]. We were thus surprised by, and initially skeptical of, the apparent paucity of Bifidobacteria in nearly all of our samples, and took steps to verify that our results were accurate. Bifidobacteria-specific qPCR corroborated the conclusion from our microarray results that Bifidobacteria were rarely major constituents of the GI microbiota, at least in this study population, and that in most babies, they did not appear until several months after birth, and thereafter persisted as a minority population. Although it is conceivable that there are geographical or demographic differences in the prevalence of Bifidobacteria, we suspect that the emphasis on Bifidobacteria in studies and reviews of the infant GI microbiota may be out of proportion to its prevalence, abundance, and relevance to health.

The results presented here suggest numerous future avenues of research. An intriguing feature of the bacterial population dynamics was the occurrence of abrupt shifts punctuating intervals of relative stability. Except in one case (the antibiotic treatment of baby 8), we could not readily identify a strong candidate for the cause of the shifts we observed. Some possibilities include bacteriophage outbreaks that can selectively decimate a dominant taxonomic group [52]; stochastic, opportunistic invasion of a metabolic or anatomic niche by a fitter species; and subtle developmental or diet-induced changes in the gut environment tipping the fitness balance in the population. Other important avenues for future research will be comparing the composition and evolution of microbial communities encountered in these healthy babies to those of preterm or otherwise unhealthy babies and to investigate the effects of antibiotics, diet, and mode of delivery on the development and evolution of these communities. Even though the healthy babies in this study assumed a large range of microbial community profiles, they were similar in several respects, most notably in the major contributing phyla, in the acquisition of certain key phyla over time, and in the relative stability of their profiles over time. It may be that in other states of health or disease, we will find either species or groups that are novel to this environment, or unusual combinations of this newly defined set of “usual” species.

Importantly, although we have shown that the gut microbiota becomes increasingly stereotyped over the first year, it is clearly established that stable interindividual differences persist even in adults [15,16]. When and how do these stable “intrinsic” characteristics of the microbiota of each individual develop? How long do they persist? How do the differing stabilities of colonization by different bacteria relate to their microanatomic (e.g., crypt vs. villus vs. mucous layer) or metabolic niches? Identifying the environmental and genetic factors that determine the distinctive characteristics of each individual's microbiota, and determining whether and how these individual specific features affect the host's physiology and health, will be an important goal for future investigations, in which the microarray described in this study will be a useful tool.

## Materials and Methods

### Microarray design and production.

The microarray contained 10,500 DNA probes (10,265 unique sequences). The probes comprised 1,379 control probes (1,144 unique sequences) and 9,121 unique taxonomically specific probes (5,938 group-level and 3183 species-level probes), consisting of 40-nucleotide (nt) sequences derived from the SSU rRNA genes, and selected for their specificity to the corresponding species or taxonomic group. The basic principles of the design are detailed in a previous report [46]. The design was based on the 2004 prokMSA SSU rDNA sequence database and phylogenetic tree [38], containing 86,453 prokaryotic SSU rDNA sequences (5,672 archaeal and 80,781 bacterial) organized into 672 archaeal and 15,765 bacterial OTUs. OTUs were defined in prokMSA as groups of sequences with identity scores (as defined in [38]) greater than either 95% or 98% (in certain medically relevant genera). We distilled this database by selecting a single high-quality sequence representative for each OTU, and trimmed the sequences to within the regions amplified by universal bacterial (Bact-8F: 5′-AGAGTTTGATCCTGGCTCAG-3′ [53] + 1391R: 5′-GACGGGCGGTGTGTRCA-3′ [54]) or archaeal (Arch344 [55]/1391R) primers. The OTUs in our pruned prokMSA database were organized into 945 (53 archaeal + 892 bacterial) taxonomic groups (nodes) containing multiple OTUs, and 92 single OTU “nodes.”

The nomenclature of the database was such that each OTU was designated by a numerical code that indicated its prokMSA taxonomy, e.g., species “1.2.3.4.5.6.007” belongs to superkingdom 1, phylum 1.2, class 1.2.3, order 1.2.3.4, family 1.2.3.4.5, genus 1.2.3.4.5.6. In this manuscript, taxonomic levels are referred to according to their depth in the OTU code, e.g., species “1.2.3.4.5.6” belongs to the more-inclusive group “1” at level 1 and less-inclusive group “1.2.3.4: at level 4.

We generated a large set of candidate probes by using BLAST [56] to predict the potential for hybridization of overlapping 40-nt sequences from each OTU in our distilled sequence database with each of the other OTUs in the database (by tiling across each sequence with window of two). A sequence was deemed a candidate probe for a specific taxonomic group if it was predicted to hybridize to at least 10% of that group's members, and not to any non-group members, using an empirically determined threshold of 28 out of 40 BLAST match–mismatches (total nucleotide matches minus mismatches to the best BLAST hit for a given rDNA sequence) as our cutoff for potential hybridization [46]. From the resulting set of candidate probes, we selected, for each node/taxonomic group, the two probes predicted to hybridize to the largest fraction of that group. We also selected probes from our candidate set such that each OTU in our distilled database was represented by probes at as many taxonomic levels as possible. Due to space limitations on the array, we were unable to print species-specific probes for every OTU in our database. Instead, we supplemented the set of group-level probes designed as described above with three additional sets of probes. First, we compiled a list of bacterial species that were either medically relevant or known human commensals. We identified the prokMSA codes for each of these species and tested the selected sequence from that OTU for species-specific 40-nt sequences, as defined by a BLAST match–mismatch score no greater than 27 to any other sequence. We also evaluated the species-specific probes from our previous array design [46] in the context of the prokMSA database, and included 467 such species-specific probes, representing 286 species. The final category of taxonomic probes were the “novel OTU” probes—316 probes designed to represent recently discovered SSU rDNA “species” that were identified in studies of the adult human colon [15] or stomach [57] (novel OTUs were defined by a 99% identity cutoff as described in the original studies). Finally, our microarray design included 1,153 control probes—positive and negative—designed for normalization and systematic examination of hybridization behaviors. The negative controls included both non-rDNA sequences and reverse complement rDNA sequences, whereas the positive controls included primer sequences and several sets of overlapping probes covering complete bacterial, archaeal, and eukaryotic SSU rRNA genes. Surface-attached oligonucleotide probes were synthesized in situ as previously described [58], with a 10-nt poly-T linker used to tether the specific 40-nt DNA probe (Agilent Technologies, http://www.chem.agilent.com). All arrays also included 307 standard Agilent control probes. All probe sequences and annotations are available in Dataset S6.

### Array coverage.

Our microarray probe set included one or more group-specific probes for 649 of the 950 taxonomic groups in prokMSA and species-specific probes for 1,590 bacterial and 39 archaeal species. Taken together, these probes ensured that 15,406 (94%) of the 16,437 species represented in the prokMSA database had at least one representative probe at some level in the tree from phylum to species, and most prokMSA species (74%) had representative probes at multiple taxonomic levels (mean of 2.4 levels per species).

### Study subjects and sample collection.

Thirteen healthy pregnant women were recruited at the Stanford University Medical Center. All study participants, including 14 babies (one set of fraternal twins), nine fathers, 13 mothers, and two siblings (1–2 y old) provided informed consent or were consented for by their parents. The study design was approved by the Stanford University Administrative Panel on Human Subjects in Medical Research. At 36–40 wk of gestation, vaginal swabs (Copan Diagnostics, http://www.copanusa.com) were obtained from ten of the 13 mothers and immediately frozen at −20 °C. After birth, infant stool samples were obtained by the parents using stool collection vials (Sarstedt, http://www.sarstedt.com/php/main.php), which contained a spoon for standardized collection of approximately 300 mg of material. Infant stool samples were collected according to the prescribed schedule (Table 1) and immediately stored in home freezers. A maternal “day 0” stool sample was obtained within 0–5 days following delivery. Stool samples were transported on ice to the laboratory for processing 2 wk, 3 mo, and 6 mo after birth of the baby. Twelve of the mothers provided breast milk samples (~20 ml) 3–9 mo after delivery, and one of them also provided breast milk 6 d after delivery; these samples were collected in 50-ml tubes and frozen immediately. Nine of the study families also provided contemporaneous stool samples from mother, father, and baby 12–17 mo after the baby's birth. Upon arrival in the laboratory, all samples were immediately transferred to a −80 °C freezer, and stored there until processing. A total of 548 samples were collected, including 471 stool samples from babies, 39 stool samples from mothers, nine stool samples from fathers, two stool samples from siblings, 16 breast milk samples, and 11 vaginal swabs. Parents were instructed to keep a journal recording key events in the categories of illness, medication, dietary change, and travel. Table 2 contains selected information for each baby (e.g., gender and method of delivery).

### DNA extraction and SSU rDNA amplification.

DNA was extracted from stool samples using the QIAamp Stool DNA mini kit (Qiagen, http://www1.qiagen.com). Vaginal swabs were processed using the QIAamp DNA mini kit (Qiagen). Milk samples were first concentrated by spinning 2 ml in a microcentrifuge for 10 min at 5,000 *g,* removing 1,800 μl of the supernatant. The pellet was resuspended in 200 μl of the remaining supernatant, and DNA was extracted using the QIAamp DNA mini kit. Samples were processed in batches of approximately 16, and multiple extraction controls were included in each run to monitor contamination. The ratio of samples to extraction controls was 6.8 for stool, 2.8 for vaginal swabs, and 5.3 for milk.

SSU rDNA was amplified from extracted DNA using broad-range bacterial-specific primers Bact-8F (5′-AGAGTTTGATCCTGGCTCAG-3′) [53] and T7-1391R (5′-AATTCTAATACGACTCACTATAGGGAGACGGGCGGTGTGTRCA-3′) [46,54]. These primers amplify approximately 90% or more of the full-length bacterial SSU rRNA coding sequence, and provide a promoter for T7 RNA polymerase. PCR mixtures were composed of 1× PCR buffer II (Applied Biosystems, http://www.appliedbiosystems.com), 1.5 mM MgCl_2_, 0.05% Triton X-100, 20 mM tetramethylammonium chloride, 2% dimethyl sulfoxide, 0.1 mM concentrations of each deoxyribonucleoside triphosphate, 0.4 μM concentrations of each primer, 2.5 U of AmpliTaq DNA polymerase (Applied Biosystems), and 5 μl of extracted DNA in a final volume of 50 μl. The PCR conditions used were 5 min at 95 °C, 20 cycles of 30 s at 94 °C, 30 s at 55 °C, and 90 s at 72 °C, followed by 8 min at 72 °C. Amplification was carried out by using a GeneAmp PCR system 9700 (Applied Biosystems). In cases of extremely low yield, multiple 20-cycle reactions were pooled. PCR reactions were cleaned up in 96-well format using Invitrogen's Charge Switch PCR Purification bead-based system (Invitrogen, http://www.invitrogen.com), and stored at −20 °C.

### Reference pool construction.

Our common reference was an equimolar mix of SSU rDNA amplicons from each sample (infant or maternal stool, vaginal, or breast milk) collected before the subject infant was 1 y old. To create the equimolar mix, purified 20-cycle PCR products were quantitated in 96-well format using the Quant-It PicoGreen dsDNA kit (Molecular Probes, http://probes.invitrogen.com) and pooled in equal amounts. The approximate fractions of stool-, vaginal-, and milk-derived SSU rDNA in the resulting pool were 90%, 5%, and 5%, respectively. This DNA pool was used both as a template for in vitro transcription (for microarray hybridizations) and for construction of a SSU rDNA clone library.

### Sequence analysis and taxonomic classification of cloned rDNA PCR products.

The reference pool (an equimolar mix of SSU rDNA amplicons from most samples, described above) was cloned and sequenced as previously described [15]. We obtained 3,458 high-quality bacterial rDNA sequences of length greater than 800 nt, including both 3,163 double reads and 295 single reads. These sequences were taxonomically classified using the Ribosomal Database Project (RDP) classifier (summarized in Table 3).

We also cloned and sequenced several hundred SSU rDNA amplicons (mean = 342; range = 125–557 adequate sequences) from each of 12 diverse individual samples in the same way, yielding a total of 4,100 high-quality sequences of length greater than 800 nt. The 12 samples sequenced consisted of ten stool samples (day 11 from baby 2; day 1 from mother of baby 3; week 12 from baby 3; month 6+ from baby 8; day 1 and day 2B from baby 10; day 12 from baby 12; month 7 from baby 13; month 7 from mother of twins: babies 13 and 14; and month 7 from baby 14), one milk sample from the mother of twins 13 and 14, and one vaginal sample from the mother of twins 13 and 14.

Each sequence from these 12 individual samples was taxonomically classified according to the 2004 prokMSA taxonomy [38] using BLAST. Specifically, we used BLAST to find the sequence with the most matches in the entire prokMSA database (also trimmed to within 8F and 1391R). The top two hits were reported and compared (hits with fewer than 600 matched nucleotides were not considered), and if these two hits mapped to the same OTU, then the sequence was classified to that OTU. If the top two hits differed in their taxonomic code, then the sequence was classified only at the most-specific level shared by the top two hits. In cases in which the second-best hit was considerably worse (matches 2nd/matches 1st < 0.9), only the best hit was considered. The prokMSA OTU codes explicitly define the taxonomic classification of a sequence at all phylogenetic levels for all of the 4,100 high-quality “individual sample” sequences.

### Labeling and hybridization.

Purified SSU rDNA amplicons were used as a template for in vitro transcription-based synthesis of amino-allyl–labeled single-stranded RNA using the MEGAScript T7 In Vitro Transcription Kit (Ambion, http://www.ambion.com). Transcription reactions were cleaned up in 96-well format using Ambion's MagMax RNA Purification system and stored at −20 °C. Large batches (5–10 μg) of reference RNA (equimolar pool of all samples; see below) were labeled using Cy3 NHS ester and stored for several weeks for repeated use. On the day of hybridization, 1 μg of each sample RNA was labeled using Cy5 NHS ester as described previously [46]. We then combined 100 ng of Cy5-labeled sample and 100 ng of Cy3-labeled reference pool in a volume of 48 μl (in nuclease-free water). We then added 2 μl of Agilent's 25× fragmentation buffer, and fragmented the RNA by heating at 70 °C for 30 min before stopping the reaction by putting it on ice and adding 50 μl of Agilent's 2× hybridization buffer. Immediately before hybridization, we heated the reaction to 95 °C for 5 min, then cooled it on ice before adding 120 μl of 1× hybridization buffer to 100 μl of fragmented, labeled RNA, and loaded 200 μl of this mixture into a hybridization chamber (Agilent). Arrays were hybridized at 60 °C in a rotating oven for 14–18 h. Slides were washed in 6× SSC, 0.005% TritonX-102 for 5 min at room temperature, then in 0.1× SSC, 0.005% TritonX-102 for 5 min, and scanned immediately using an Agilent DNA Microarray Scanner. Washing and scanning were performed in a low-ozone environment [59].

### Microarray data normalization.

Data were extracted from the scanned microarray image using the most current version of the Agilent Feature Extraction software (Versions 7.1.1–8.1.1). The raw background-subtracted Cy5 (or Cy3) fluorescence intensity values for each probe were normalized by dividing the Cy5 (or Cy3) values by the median Cy5 (or Cy3) value of the universal probe “UNIV2” (extended version of 3′ PCR primer 1391R: 5′-GTGGGGAGCGAACAGGATTAGATACCCTGGTAGTCCACGC-3′) from the corresponding array, and multiplying by 100. At this stage, values ranged from 0.01 to approximately 100; values greater than 100 were rare, occurring only when the fluorescent signal for a specific probe was brighter than that of the universal probe. The normalized Cy5 values were “decompressed” by the following correction: decompressed Cy5 equals 10 to the power of log_5_ of the normalized Cy5 intensity. This decompression corrects for the nonlinear relationship of the hybridization signals to the relative abundance of the target species, which we observed in a series of serial dilution experiments described in a previous report [46]. In those experiments, we found that a 10-fold change in abundance translated into approximately a 5-fold change in the corresponding fluorescence intensity. Following this transformation, the expanded range of values was 0.001 to approximately 700. We used BLAST to predict the hybridization of each of the 3,458 common reference rDNA sequences greater than 800 base pairs, using a weighting scheme described previously [46], such that a probe with a perfect match to every sequence would have an expectation of 100%, and probes with fewer or imperfect matches to the sequences in the reference pool would have correspondingly lower expected hybridization values. We then calculated the log (observed/expected) ratio for each probe in the context of our pooled sample reference mix and applied these probe-specific correction factors to the decompressed Cy5 values.

### Microarray probe filtering.

Microarray analysis of synthetic pools of defined composition allowed us to identify poorly performing probes, which were then excluded from further analyses. We evaluated probe performance using five synthetic pools (four pools of six unique rDNA PCR products, one pool of 230 unique rDNA PCR products [from [46]]) and one biological pool (the common reference described above), whose composition was characterized by deep sequencing. Probes with fluorescence intensity values greater than 0.5% of that of the universal probes despite the lack of predicted sequence homology (defined as a BLAST match–mismatch value less than 25 out of a possible 40) to any of the species in the sample were excluded from subsequent analyses. We also discarded data from probes that had an observed (decompressed) signal intensity 100-fold higher or lower than expected, in the biological reference pool analyses, (as described above in the Microarray data normalization section). The remaining set of 6,381 well-behaved probes was used in all subsequent analyses.

### Estimation of taxonomic group abundance.

For each sample, we derived an estimate of the relative abundance of each taxonomic group in our phylogenetic tree using an algorithm that ensures that no species contributes more than once to the estimate of a taxonomic group abundance, and that the downstream probes (probes that represent distinct subsets of species belonging to that phylogenetic group) are incorporated into the cumulative group abundance estimate. Specifically, for each phylogenetic group in each sample, we sorted all of the downstream probes according to their microarray-based relative abundance estimates and calculated the total sum of the relative abundance estimates of all nonoverlapping probes. As a result, the specific probes added together to represent a given taxonomic group varied across samples, depending on which specific probes had the greatest hybridization signal in that sample.

### Comparison of microarray and sequence data.

Estimates of the relative abundance of each prokMSA group, at each level of the taxonomic hierarchy, in the reference pool and in the 12 individual samples analyzed by sequencing, were derived by calculating the proportion of the sequences in the corresponding rDNA library that were assigned by BLAST to that group. These sequence-based abundance estimates were directly compared to those derived from the microarray data as described in the previous section.

### Autocorrelation analysis.

We used autocorrelations as a way to measure the tendency of a taxonomic group to persist once established (“stickiness”). For a given time series from time *a* to time *b,* we calculated the Pearson correlation for each baby of the vector (*a* + 1,…, *b*) and vector (*a,*…, *b* − 1) representing the log(relative abundance) estimates. The autocorrelation of each taxonomic group was then taken to be the median autocorrelation across all of the babies for which the taxonomic group in question was present at least once (defined as abundance >0.1%) in the time interval in question. In our “stickiness” analysis, we performed this analysis separately for two different sets of samples, collected at different sampling intervals, in order to avoid the confounding effects of variation in sampling intervals: (1) once weekly samples from weeks 1–12; and (2) monthly samples from months 1–6.

### Detection of archaeal and fungal sequences.

To screen for archaeal and fungal rDNA sequences. we first screened pools comprising all of the extracted DNA samples from each baby (i.e., one pool per baby), all parental stool samples, all vaginal samples, and all milk samples, respectively. For each pool that gave a positive result, all the component samples were then analyzed individually. To screen for archaeal rDNA sequences, we used two sets of broad-range archaeal-specific primers: A751F and U1406R [60]; and Arch333F (5′-TCCAGGCCCTACGGG-3′ [61]) and Arch958R (5′-YCCGGCGTTGAMTCCAATT-3′ [62,63]). To screen for fungi, we used the broad-range fungal-specific primers 817F [64] and 1536R-rev (5′-AATRCAATGCTCTATCCCCA-3′, adapted from [64]). PCR mixtures were similar to those used for the broad-range bacterial PCR described above, but with the following changes: for the fungal-specific PCR, the MgCl_2_ concentration was increased to 2 mM, and BSA was added in a final concentration of 1 mg/ml. To both fungal-specific and archaeal-specific PCR reactions, no tetramethylammonium chloride was added, and 2 μl of pooled DNA was added, with a final volume of 50 μl. The cycling program consisted of 5 min at 95 °C, 35 cycles of (30 sec at 94 °C, 30 sec at 55 °C, and 30 sec at 72 °C), followed by 8 min at 72 °C. Amplification reactions were analyzed on agarose gels.

### Analysis of amplification bias.

To investigate whether mismatches to the broad-range bacterial primers could affect amplification, DNA was extracted from five bacterial reference strains: Escherichia coli (TOP10 cells, Invitrogen), Clostridium perfringens (ATCC 13124), Bacteroides fragilis (ATCC 25285), Bifidobacterium longum (ATCC 15707), and Bifidobacterium infantis (ATCC 15697), using the QIAamp DNA mini kit. DNA concentrations were measured using a UV spectrophotometer, and adjusted to correct for DNA yield, genome size, and number of SSU rRNA gene copies per genome to obtain “normalized” DNAs, each containing the same number of SSU rRNA gene copies per μl. Bacterial DNA from lysates was amplified individually or in pairs, using primers 8F and T7-1391R as described above, using either 20 or 35 cycles. For the paired reactions, normalized Bifidobacterium longum DNA was mixed with undiluted or serial dilutions of normalized DNA from *Escherichia coli, Clostridium perfringens,* or Bacteroides fragilis. After amplification, PCR mixtures were purified using the QIAquick PCR purification kit (Qiagen) and digested using a set of restriction enzymes selected to distinguish between the two PCR products obtained with the paired species. Digestions were analyzed on agarose gels, to quantitate the relative abundance of the PCR products representing Bifidobacterium longum and the species in the comparison group, respectively.

### Quantitative PCR.

A separate real-time qPCR assay was used to amplify and quantify rDNA from each of four microbial groups: total bacteria, Bifidobacteria, total fungi, and total archaea. Total bacterial qPCR was performed using a 10:1 mixture of the universal forward primer 8FM (5′-AGAGTTTGATCMTGGCTCAG-3′, adapted from [53]) and corresponding Bifidobacterium longum forward primer 8FB (5′-AGGGTTCGATTCTGGCTCAG-3′, this study), with reverse primer Bact515R (5′-TTACCGCGGCKGCTGGCAC-3′, adapted from [54]) and TaqMan probe Bact338K (5′-FAM/CCAKACTCCTACGGGAGGCAGCAG/TAMRA-3′, adapted from [65]). We supplemented the universal bacterial forward primer with the Bifidobacterium longum forward primer because an analysis of SSU rDNA sequences showed that this group was an outlier in that it had three mismatches to our forward primer 8FM. Pilot studies showed that this primer mixture allowed comparable amplification of SSU rRNA genes from representative Bifidobacteria, *Bacteroides,* Enterobacteria, and Clostridia. *Bifidobacterium* genus qPCR was performed using primers Bif42F [26] and Bif164R (5′-CATCCGGCATTACCACCCGTT-3′, adapted from [66]), and probe Bif126_Taqman [26]. Total fungal qPCR was performed using primers ITS1F-F (5′-CTTGGTCATTTAGAGGAAGTAA-3′ [67]) and ITS4-R (5′-TCCTCCGCTTATTGATATGC-3′ [68], and TaqMan probe 5.8S (5′-FAM/CATTTCGCTGCGTTCTTCATCGATG/TAMRA-3′, adapted from [68]. Archaeal qPCR was performed using primers Arch333F (5′-TCCAGGCCCTACGGG-3′) [61] and Arch958R (5′-YCCGGCGTTGAMTCCAATT-3′) [63], and TaqMan probe 515F (5′-FAM/GTGCCAGCMGCCGCGGTAA/TAMRA-3′, adapted from [54]). For all qPCR assays, each 20-μl reaction contained 1× TaqMan Universal PCR master mix (Applied Biosystems), 0.9 μM of each primer (0.09 μM of primer 8FB in the total bacterial assay), 0.2 μM of the probe, 1 U of AmpliTaq Gold DNA polymerase (Applied Biosystems), and 2 μl of extracted DNA. The thermal cycling program consisted of 95 °C for 10 min, followed by either 40 cycles (bacterial and bifidobacterial assays) or 45 cycles (fungal and archaeal assays) of 95 °C for 30 s, 55 °C for 30 s, 60 °C for 45 s, 65 °C for 15 s, and 72 °C for 15 s [69]. Reactions were carried out in a Prism 7900HT Sequence Detection System (Applied Biosystems). Ten-fold serial dilutions of known quantities of rDNA from the appropriate microbial group (i.e., bacteria, Bifidobacteria, fungi, or archaea) were used to generate standard curves. Absolute rDNA abundance was calculated based on the standard curves using SDS software version 2.1 (Applied Biosystems) with the baseline set at cycles 3–15 (bacterial and bifidobacterial assays) or cycles 3–13 (fungal and archaeal assays), and the cycle threshold set within the geometric phase of the amplification curve. Sensitivity of each assay was approximately 100 rDNA molecules per PCR reaction well. Every qPCR reaction plate included two types of negative controls (reagent control and aliquot control), each in triplicate. Specificity of the bifidobacterial real-time PCR assay was tested using genomic DNA extracted from 17 bacterial reference strains: Bacillus subtilis (ATCC 6633), Bacteroides fragilis (ATCC 25285), Bacteroides thetaiotaomicron (ATCC 29148), Bifidobacterium longum (ATCC 15707), Bifidobacterium infantis (ATCC 15697), Clostridium perfringens (ATCC 13124), Clostridium putrefaciens (ATCC 25786), Enterococcus faecalis (ATCC 19433), Escherichia coli (TOP10 cells; Invitrogen), Haemophilus haemolyticus (ATCC 33390), Lactobacillus acidophilus (ATCC 4356), Lactobacillus delbrueckii (ATCC 4797), Megasphaera elsdenii (ATCC 17752), Proteus vulgaris (ATCC 13315), Pseudomonas aeruginosa (ATCC 10145), Staphylococcus aureus (ATCC 25923), and Streptococcus salivarius (ATCC 13419).

### Validation of suspect species/taxa.

We investigated the origin of array hybridization signals representing 12 species/taxa whose presence in the samples was unexpected and uncorroborated by sequences in the reference pool. For each analysis, we used one or both of two independent assays. First, we attempted to amplify the sequences apparently detected by the array analysis, using species/taxa specific primers; when a product was obtained, it was further analyzed by sequencing. To amplify the sequences, we used a primer identical to the 40-mer probe that yielded a hybridization signal in our microarray as the 5′ primer and a 40-mer universal sequence (the reverse complement of sequence UNIV2 given above) as the 3′ primer. In some cases, we also tried to amplify the suspect sequence using a truncated (23-mer) version of the corresponding oligonucleotide probe from the microarray paired with a known group-specific PCR primer from ProbeBase [55]. All PCRs were performed under conditions identical to those used in the original amplifications of samples for microarray analysis. Positive bands of expected size were cloned and sequenced. As a second approach, four samples predicted from the microarray results to contain sequences from unexpected species were further analyzed by sequencing of SSU rDNA clone libraries (96–288 clones), generated by amplification using broad-range bacterial primers 8F and 1391R (as above), and cloning and sequencing as previously described [15]. The relative abundances predicted by microarray analysis and the numbers of clones sequenced were as follows: *Vibrio* (13%): 96, *Deinococcus* (0.1%): 192, *Spirochaetes* (1%): 288, and Legionella pneumophila (1%): 192.

## Supporting Information

Dataset S1 The prokMSA Sequence Database (2004)—Fasta File (26.4 MB GZ).

Dataset S2 Phylogenetic Tree for 2004 prokMSA Sequence Database (5.0 MB TXT)

Dataset S3 Normalized Microarray Data for All Samples and All Filtered Probes Raw Cy5 intensities were transformed as described in Materials and Methods. Note that for samples with replicate hybridizations (*n* = 36), averaged values are shown, and sample names are indicated by (Avg).
(19.0 MB TXT)

Dataset S4 Microarray-Derived Relative Abundance for All Samples and All Well-Measured Taxonomic Groups/Species Relative abundances were estimated from normalized Cy5 intensities of all filtered probes as described in Materials and Methods.
(4.7 MB TXT)

Dataset S5 Quantitative PCR Results for Three Kingdoms Results from qPCR measurements of total bacteria, fungi, archaea, and Bifidobacteria for all samples.
(404 KB XLS)

Dataset S6 Microarray Probe Annotations (2.4 MB XLS)

Figure S1 Abundance of Bacteria and Bifidobacteria throughout the First Year of Life For each baby sample, abundance of total bacteria and Bifidobacteria were estimated by TaqMan real-time PCR as detailed in Materials and Methods. For each of these taxonomic groups, estimated rRNA gene copies per gram of feces (*y*-axis) are plotted as a function of days of life (*x-*axis). Both axes are on a logarithmic scale. For each taxonomic group, the abundance measurements are truncated on the lower end of the range at the value corresponding to the 95th percentile of the extraction (negative) controls for the corresponding set of PCR primers (10,702 and 1,183 rDNA copies per gram of stool equivalent for total bacteria and Bifidobacteria, respectively). Thus, values plotted at the corresponding lower detection limits generally represent abundances below the indicated value (a range that includes zero).
(457 KB PDF)

Figure S2 Abundance of Fecal Bacteria, Fungi, and Archaea throughout the First Year of Life For each baby sample, abundance of bacteria, fungi, and archaea was estimated by TaqMan real-time PCR as detailed in Materials and Methods. For each of these taxonomic groups, estimated rRNA gene copies per gram of feces (*y*-axis) are plotted as a function of days of life (*x*-axis). Both axes are on a logarithmic scale. For each taxonomic group, the abundance measurements are truncated on the lower end of the range at the value corresponding to the 95th percentile of the extraction (negative) controls for the corresponding set of PCR primers (10,702, 9,831, and zero rDNA copies per gram of stool equivalent for bacteria, fungi, and archaea, respectively). Thus, values plotted at the corresponding lower detection limits generally represent abundances below the indicated value (a range that includes zero). All values for which the qPCR estimated abundance value was zero (archaea only) were omitted. Episodes of antibacterial or antifungal (nystatin) treatment are indicated on the temporal axis by gray or pink bars, respectively (see Table 1 for additional information).
(773 KB PDF)

## Abbreviations

GI: gastrointestinal
nt: nucleotide
OTU: operational taxonomic unit
prokMSA: prokaryotic multiple sequence alignment
qPCR: quantitative PCR
rDNA: ribosomal DNA
rRNA: ribosomal RNA
SSU: small subunit
