# Supplementary material for: Development of the Human Infant Intestinal Microbiota
Source: PLoS Biol. 2007 Jun 26;5(7):e177. doi: 10.1371/journal.pbio.0050177 (PMC1896187; doi:10.1371/journal.pbio.0050177)
Supplement: Supplementary file 1 [file pbio.0050177.sg001.pdf]

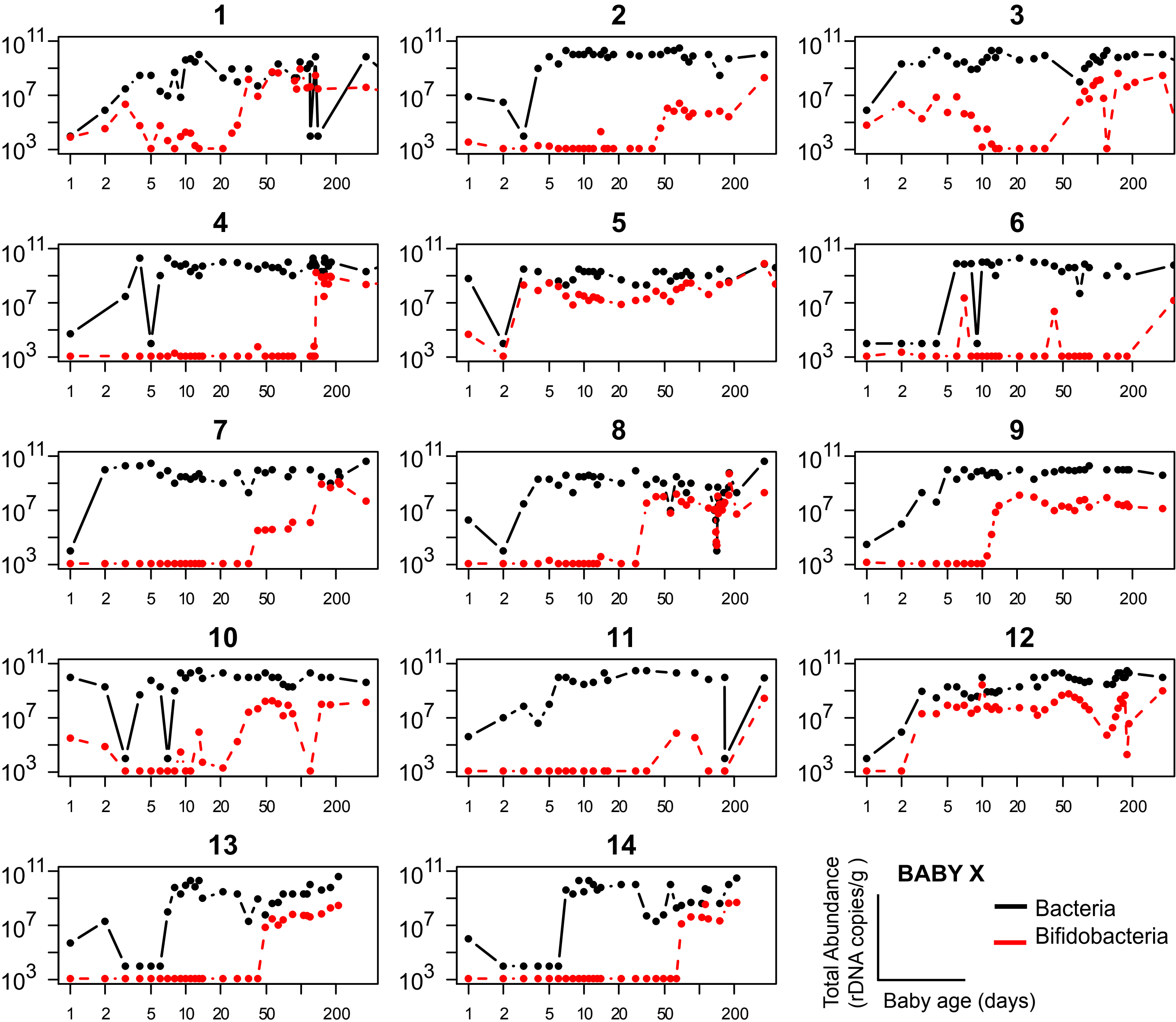
1
2
3
4
5
6
7
8
9
10
11
12
13
14
10^{11}
10^{7}
10^{3}
1 2 5 10 20 50 200
BABY X
Total Abundance (rDNA copies/g)
Baby age (days)
Bacteria
Bifidobacteria
