# Supplementary material for: Development of the Human Infant Intestinal Microbiota
Source: PLoS Biol. 2007 Jun 26;5(7):e177. doi: 10.1371/journal.pbio.0050177 (PMC1896187; doi:10.1371/journal.pbio.0050177)
Supplement: Supplementary file 2 [file pbio.0050177.sg002.pdf]

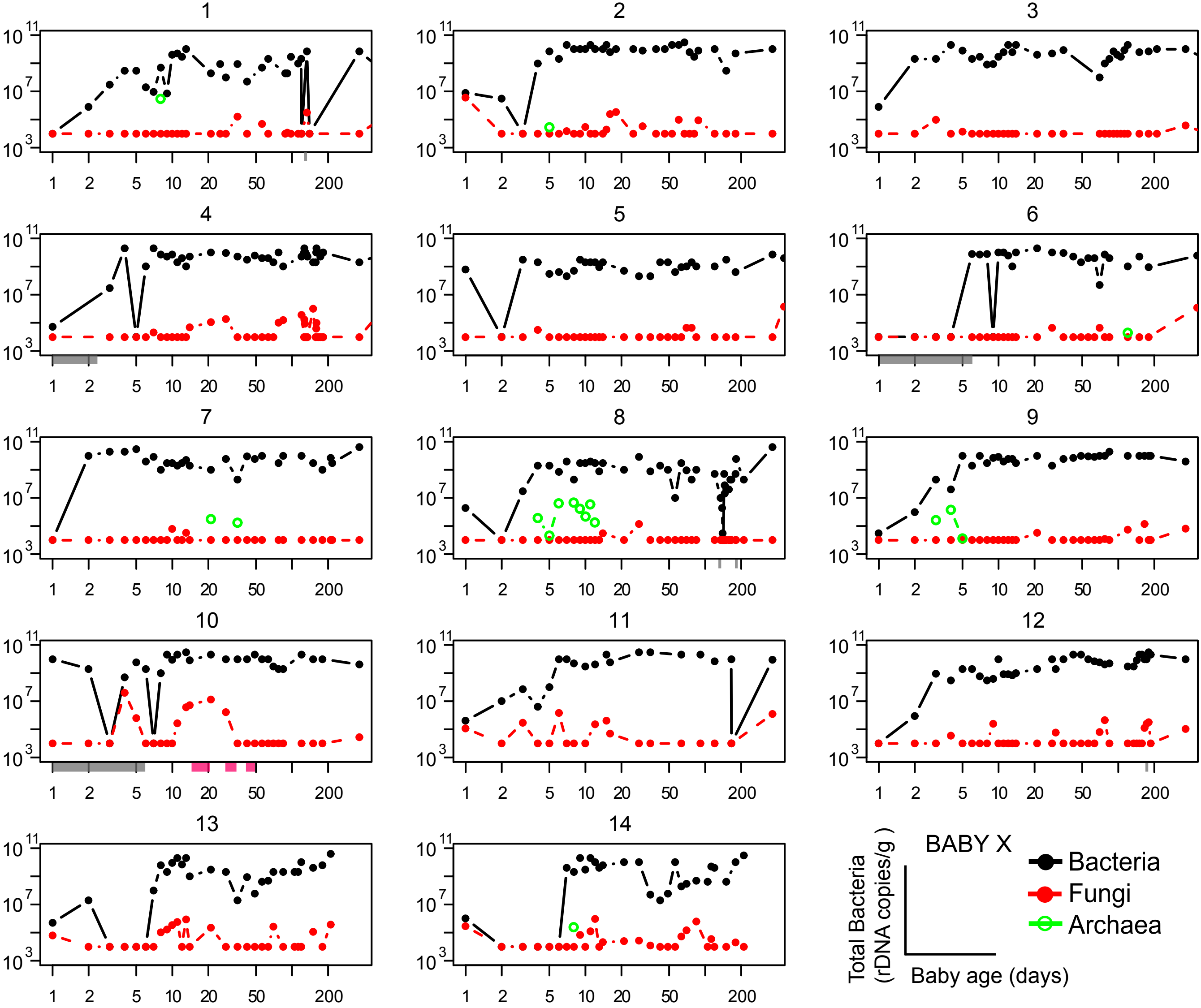

1
2
3
4
5
6
7
8
9
10
11
12
13
14
$10^{11}$
$10^{7}$
$10^{3}$
1 2 5 10 20 50 200
BABY X
Total Bacteria (rDNA copies/g )
Baby age (days)
Bacteria
Fungi
Archaea
